# Developing Stem Cell-Based Therapeutic Strategies in Orthopaedic Surgery

**DOI:** 10.1155/2018/5704192

**Published:** 2018-04-26

**Authors:** Kivanc Atesok, Mitsuo Ochi, Nicola Baldini, Emil Schemitsch, Meir Liebergall

**Affiliations:** ^1^Department of Orthopaedic Surgery, University of Alabama at Birmingham, Birmingham, AL, USA; ^2^Department of Orthopaedic Surgery, Hiroshima University, Hiroshima, Japan; ^3^Department of Biomedical and Neuromotor Sciences, The Rizzioli Institute, University of Bologna, Bologna, Italy; ^4^Department of Surgery, Western University, London, ON, Canada; ^5^Department of Orthopaedic Surgery, The Hadassah-Hebrew University Medical Center, Jerusalem, Israel

The term “stem sell” first appeared in scientific literature in 1868, when the German scientist Ernst Haeckel merged the concepts of phylogeny and ontogeny to describe the “stammzelle” (stem cell), an evolutionary concept of a primordial cell that evolves into all cells and multicellular organisms [1]. Although Haeckel's argument and use of the term “stem cell” nearly 150 years ago were impressive, they were based on his observations of embryo development and the distinction between fertilized and nonfertilized eggs. The first definitive evidence for the existence of stem cells came in the early 1960s, after the Canadian scientists Ernest McCulloch and James Till performed experiments on the bone marrow of mice and observed that different blood cells come from a special class of cells [2]. Stem cell research has progressed rapidly since 1981, when the British scientist Martin Evans and the American scientist Gail Martin succeeded in isolating and culturing mice embryonic stem cells (ESCs). In the late 1990s, American scientist James Thomson first isolated human ESCs, revealing the potential for a pluripotent stem cell that would be a source of various germ layers and new organs [3]. Arguably, this recognition of the pluripotency of human stem cells triggered the exponential developments in stem cell research seen over the last two decades. Regenerative medicine has emerged as a new discipline, and its principles have been extended to surgical and nonsurgical specialties in medicine.

As a surgical specialty covering the pathologies of tissues and organs derived from two different germ layers, including the mesoderm (bone, cartilage, muscle, and ligaments) and the ectoderm (spinal cord and nerves), orthopaedic surgery is arguably one of the disciplines in which stem cell-based therapeutic strategies can be applied the most diversely.

This special issue is aimed at presenting and discussing the current concepts and groundbreaking developments in stem cell-based treatment strategies in the field of orthopaedic surgery. Since adipose tissue is easier to retrieve and can often be harvested in ample quantities, K. Kjaergaard et al. studied the in vivo osteogenic potential of adipose-derived culture-expanded stem cells in an animal model. The authors showed that adipose-derived cells were capable of forming new bone, although their potential was significantly lower than that of stem cells derived from bone marrow. C. L. Roberts et al. aimed to answer the question of whether human osteoblasts can be reverted to the pluripotent embryonic state. Their results demonstrated that pluripotent stem cells that are reverse induced from osteoblasts are capable of generating osteogenic and chondrogenic cells, but lack the ability to form adipocytes.

N. Kamei et al. reviewed endothelial progenitor cells (EPCs) as a promising source of cell-based therapies to regenerate musculoskeletal and neural tissues. B. D. Bates et al. demonstrated the potential of EPCs to enhance the radiographic and morphometric parameters of bone healing when applied three weeks after bony injury in a rat model of delayed fracture healing.

Stem cell-based therapeutic applications have become the focus of intensive research in the treatment of degenerative joint pathologies, including osteoarthritis and degenerative disc disease (DDD) [4]. H. Madry et al. contributed a review article to this special issue that summarizes the technical, clinical, and biological aspects of using bone marrow aspirate concentrate with mesenchymal stem cells (MSCs) to repair and regenerate osteochondral defects. Furthermore, to enhance structure-specific regeneration of hyaline cartilage in a rabbit model, W. Guo et al. loaded MSCs onto a composite scaffold that mimicked the aligned configuration of native cartilage tissue. In another interesting study from Japan, E. E. Mahmoud et al. investigated the therapeutic potential of human multilineage differentiating stress-enduring cells for repairing osteochondral defects in an immuno-deficient rat model.

Stem cell-based therapies for DDD still struggle with challenges, including the detrimental effects of the hypoxic, inflammatory microenvironment of intervertebral discs, which reduces the activity and vitality of transplanted stem cells [4]. Realizing these challenges, W. Wang et al. investigated the effects of hypoxic preconditioned bone MSCs on repairing and regenerating intervertebral discs in a rat DDD model.

The anterior cruciate ligament (ACL) and rotator cuff (RC) are among the most frequently encountered ligament/tendon injuries in orthopaedic clinical practice [5]. However, the potential of the ACL and RC to self-regenerate and heal is recognized as extremely poor [6]. In a review article, K. J. Lee et al. summarized critical aspects of current research, focusing on the use of ligament-derived stem cells as a regenerative treatment strategy for patients with tendon/ligament pathologies.

In the field of regenerative medicine in general and in the use of stem cell-based therapeutic strategies in orthopaedic surgery in particular, one of the most pressing issues is to translate the enormous quantity of research into clinical practice. Despite the ever-increasing global activity and an accumulating body of literature, very few cell-based therapies for musculoskeletal disorders have been effectively translated into clinical practice [7, 8]. The foundation for solving this translation conundrum will be provided by orthopaedic surgeon-scientists with in-depth understanding of basic and clinical research principles. With this in mind, we gathered a guest editorial team from among the most prominent orthopaedic surgeon-scientists around the world who were willing to exercise particular attention to promote clinical research papers in this special issue. One such paper, by D. Granchi et al., investigated changes in biochemical bone turnover markers according to the progression of bone healing induced by autologous MSCs combined with biphasic calcium phosphate in patients having delayed union or nonunion of long bone fractures. Finally, the issue includes a multicentric randomized clinical trial protocol from E. Gómez-Barrena et al. and investigators from centers in Italy, Germany, France, and Spain, who aim to compare the efficacy of treatment with autologous human bone marrow-derived mesenchymal stromal cells versus iliac crest autograft to enhance bone healing in patients with diaphyseal and/or metaphysodiaphyseal fractures (femur, tibia, and humerus) that had statuses of atrophic or oligotrophic nonunion.

We hope that this special issue, “Developing Stem Cell-based Therapeutic Strategies in Orthopaedic Surgery,” will help the scientific community find answers to some of its questions and will make a few strides toward benefitting our patients.
